# Principles behind SLE treatment with *N*-acetylcysteine

**DOI:** 10.1097/IN9.0000000000000010

**Published:** 2022-10-25

**Authors:** Sandy Nasr, Andras Perl

**Affiliations:** 1 Department of Medicine, College of Medicine, State University of New York, Syracuse, NY, USA

**Keywords:** systemic lupus erythematosus, *N*-acetylcysteine, mechanistic target of rapamycin, mitochondrial hyperpolarization

## Abstract

Systemic lupus erythematous (SLE) is a multisystem chronic autoimmune disease in which disrupted molecular pathways lead to multiple clinical manifestations. Currently approved treatments include hydroxychloroquine, some immunosuppressive medications, and some biologics. They all come with a range of side effects. *N*-acetylcysteine (NAC) is an antioxidant that has shown potential benefits in SLE patients without having major side effects. The following review highlights the molecular mechanisms behind the therapeutic effect of NAC in SLE patients. A higher-than normal mitochondrial transmembrane potential or mitochondrial hyperpolarization (MHP) was found in lymphocytes from SLE patients. MHP is attributed the blocked electron transport, and it is associated with the depletion of ATP and glutathione and the accumulation of oxidative stress-generating mitochondria due to diminished mitophagy. Comprehensive metabolome analyses identified the accumulation of kynurenine as the most predictive metabolic biomarker of lupus over matched healthy subjects. Cysteine is the rate-limiting constituent in the production of reduced glutathione, and it can be replaced by its precursor NAC. Kynurenine accumulation has been reversed by treatment with NAC but not placebo in the setting of a double-blind placebo-controlled clinical trial of 3-month duration. Mitochondrial oxidative stress and its responsiveness to NAC have been linked to systemic inflammation, gut microbiome changes, and organ damage in lupus-prone mice. Given the unique safety of NAC and chronicity of SLE, the clinical trial of longer duration is being pursued.

Systemic lupus erythematous (SLE) is a multisystem chronic autoimmune disease in which every organ and tissue might be affected. A complex interplay of disrupted molecular pathways leads to the multiple clinical manifestations reflecting the diversity of organ involvement in this disease ^[1]^. A multitude of genetic, environmental and hormonal factors pave the way for its development. One of this disease’s hallmarks is the production of several autoantibodies ^[2]^. A lack of understanding of the complete pathogenesis of SLE has led to the lack of great advancements in its treatment. Currently, hydroxychloroquine remains the cornerstone in therapy due to the established benefits and acceptable safety profile. Other alternatives include immunosuppressive medications (glucocorticoids, mycophenolate, cyclophosphamide, cyclosporine) and biologic agents (eg, rituximab, belimumab). However, these carry their own side effects ^[3]^. Hence came the need to study potential non-immunosuppressive drugs like *N*-acetylcysteine (NAC).

A commonly agreed upon the fact in the pathogenesis of SLE is the dysfunction of T cells, B cells, dendritic cells, macrophages, and neutrophils, likely triggered by genes and/or the environment ^[4]^. In fact, even though autoantibodies are produced by B cells, it is well known now that T cells critically participate in the pathogenesis of SLE because of their ability to guide B cells in autoantibody production. Hence, studies have been focusing on understanding of the intrinsic abnormalities in T cells that lead to B-cell dysfunction ^[5]^. One of those findings is the abnormal activation and processing of cell death signals by the immune system which in turn lead to necrosis of the cells and stimulate the production of antinuclear antibodies in the process. In a study done by Gergely et al ^[6]^, it was found that mitochondria which are the organelles that control the signal processing of T cells are dysfunctional in lupus patients, exhibit a higher-than-normal mitochondrial transmembrane potential (ΔΨm) or mitochondrial hyperpolarization and are ATP (adenosine triphosphate) depleted which predispose to cell death by necrosis instead of apoptosis. The process of necrosis activates macrophages and dendritic cells and enhances their capacity to produce nitric oxide and interferon alpha in SLE. It was also found in this study that reactive oxygen intermediates (ROIs) are higher in lupus patients compared with healthy controls. Mitochondrial ROI production and the increase in membrane potential or mitochondrial hyperpolarization (MHP) are early checkpoints in Fas- and H_2_O_2_-induced cell death ^[7]^. In addition to that, reduced levels of glutathione were found in freshly isolated peripheral blood lymphocytes (PBLs) from lupus patients, which was consistent with ongoing oxidative stress in vivo. The deficiency of glutathione predisposes cells for ROI-induced cell death ^[6]^. Protection against ROI-mediated cell death is dependent on the availability of reduced glutathione.

A study done on murine Lupus models by Chen et al showed that the NAD^+^-modulating ectoenzyme CD38 regulates mitochondrial fitness in SLE CD8^+^ T cells and negatively affects their function and ability to combat infection. CD38 reduces cellular NAD^+^ levels, and therefore suppresses mitophagy by inhibiting the recruitment of damaged mitochondria to the phagophore through the SIRT1-PINK1-Parkin pathway. These events result in the accumulation of damaged depolarized mitochondria in CD38hiCD8^+^ T cells and reduce their ability to clear viruses effectively. These data argue strongly that mitochondria are dysfunctional in SLE T cells ^[8]^.

In another study, it was found that T cells of SLE patients show robust activation of the mechanistic target of rapamycin (mTOR), which is a sensor of the change in membrane potential of the mitochondria and therefore a key player in T-cell signaling that is defective in SLE. The relative depletion of glutathione is thought to activate mTOR ^[9,10]^. mTOR is also activated outside the immune system, and it thus mediates end-organ damage, including the kidney ^[11]^ and the liver ^[12]^.

Cysteine is the rate-limiting constituent in the production of reduced glutathione, and it can be replaced by its cell permeable precursor, *N*-acetylcysteine ^[13]^. Commercially available since long time, NAC is relatively safe and inexpensive medication. This drug is not naturally found, but cysteine is present in some foods such as chicken, garlic, and yogurt. When ingested, NAC activates the biosynthesis of glutathione which helps in detoxification and elimination of free radicals due to its powerful antioxidant properties ^[14]^.

In a study done by Suwannaroj et al ^[15]^, it was found that SLE mice treated with NAC had a significantly lower anti-DNA antibody production at 24 weeks compared to control mice and had a modest improvement in mortality ^[15]^. Shi et al linked F-actin over-polymerization to increased ROI production apoptosis, and aberrant migration of bone marrow mesenchymal stem cells (MSCs) from SLE patients ^[16]^. NAC treatment made F-actin more orderly and migration of SLE MSCs in vitro. Of note, oral administration of NAC also reversed MSC abnormalities and markedly reduced serum autoantibody levels and ameliorated lupus nephritis (LN) in MRL/lpr mice (Table 1).

**Table 1:** Preclinical studies showing clinical efficacy of NAC in SLE.

Study Type	Year of Publish	Study Model	Study Purpose	Study Population	Results	Reference
Antioxidants suppress mortality in the female NZB × NZW F1 mouse model of systemic lupus erythematosus (parallel study)	2001	Mice	Examine immunomodulatory effects of NAC and cysteamine on autoimmune disease, glomerulonephritis, and mortality in the female SLE mice	Female NZBxNZW F1 (B/W) SLE mice	NAC significantly suppressed anti-DNA antibody levels and improved mortality in the female mouse model of SLE	^[15]^
NAC effects in mice in vivo and human cells in vitro	2014	Mice	Examine NAC effect on ROI production and F-actin polymerization in SLE	MRL/lpr mice	NAC reduced autoantibody production and nephritis	^[16]^
NAC effects on lupus in MRL mice	2019	Mice	Examine NAC effect on liver injury	MRL	NAC reduced hepatitis	^[17]^
NAC effects on gut microbiome in SLE mice	2021	Mice	Examine NAC effect on oxidative stress	MRL/lpr	NAC corrected microbiome skewing and blocked autoimmunity	^[18]^

NAC, *N*-acetylcysteine; ROI, reactive oxygen intermediate; SLE, systemic lupus erythematosus.

Trichloroethene (TCE) exposure has been implicated in the development of autoimmunity, including autoimmune hepatitis (AIH) and SLE. TCE-induced antinuclear antibodies (ANA) and the formation 4-hydroxynonenal (HNE)-modified immune complexes in the bloodstream of lupus-prone MRL mice ^[17]^. Moreover, TCE triggers prominent lobular inflammation and hepatocellular proliferation in the liver of MRL mice, which were abrogated by treatment with NAC. Furthermore, TCE dramatically increased lymphocytic infiltration by T_H_17 and B cells and triggered a profound loss of regulatory T cells (Tregs) in the liver. Of note, TCE-mediated skewing of hepatic and splenic immune lineage development was effectively reversed by NAC (Table 1). Remarkably, NAC also blocked microbiome changes along with systemic autoimmunity in lupus-prone MLR/lpr mice ^[18]^, suggesting that gut dysbiosis is driven by oxidative stress on the organismal levels (Table 1).

Based on GSH depletion in patients with SLE ^[6]^, a 3-month phase I-phase II double-blind placebo-controlled randomized pilot study of NAC in 36 subjects was done to look for its immunological and therapeutic impact (Table 2). The study found the drug to be safe and effective at doses of 2.4 and 4.8 g/day in reversing the depletion of glutathione and in improving disease activity and the fatigue level. This dose of NAC reduced the SLEDAI (SLE Disease Activity Index) score and the BILAG (British Isles Lupus Assessment Group) score and profoundly reduced mTOR activity in T lymphocytes ^[9]^. Specifically, the double negative T cells are the main cells affected by the blockade of mTOR by NAC ^[9]^. In fact, kynurenine’s accumulation plays a role in the activation of mTOR in SLE ^[19]^. Kynurenine is a metabolite of the pentose phosphate pathway which serves as a metabolic checkpoint in the pathogenesis of SLE in double negative T cells which are a source of interleukin 4, interleukin 17 and necrotic debris. Treatment with NAC increased the abundance of NADPH which in turn resulted in increased catabolism by NADPH-dependent kynurenine hydroxylase leading to lower levels of kynurenine which subsequently inhibited the mTOR pathway in those T cells ^[19]^.

**Table 2:** Clinical studies involving the use of NAC in SLE subjects.

Study type	Year of publish	Study model	Study purpose	Study population	Results	Reference
Improvement in endothelial dysfunction in patients with systemic lupus erythematosus with *N*-acetylcysteine and atorvastatin (randomized controlled trial)	2011	Humans	Study the effects of NAC and atorvastatin on endothelial dysfunction in patients with SLE	Thirty-two SLE patients and age, sex-matched 10 healthy control subjects. The patients were between 17 and 65 years old	There was reduction in reflection and stiffness indices and SI and CRP marker with treatment of NAC and atorvastatin suggesting improvement in endothelial dysfunction	^[20]^
*N*-acetylcysteine reduces disease activity by blocking mammalian target of rapamycin in T cells from systemic lupus erythematosus patients (randomized, double-blind, placebo-controlled trial)	2012	Humans	Examine the safety, tolerance, and efficacy of the glutathione (GSH) precursor NAC	36 SLE patients	NAC safely improves lupus disease activity (improve SLEDAI and BILAG scores) by blocking mTOR in T lymphocytes	^[9]^
Attention-deficit and hyperactivity disorder scores are elevated in response to NAC treatment in patients with systemic lupus erythematosus (randomized controlled trial)	2013	Humans	Investigate whether 80HD may serve as a marker of neuropsychiatric disease and as a target for NAC treatment in patients with SLE	49 patients with SLE and 46 matched healthy control subjects	ADHD Self Report Scale scores were higher in patients with SLE and they were lowered by NAC treatment	^[21]^
Increased Mitochondrial Electron Transport Chain Activity at Complex I Is Regulated by *N*-Acetylcysteine in Lymphocytes of Patients with Systemic Lupus Erythematosus (randomized controlled trial)	2014	Humans	Determine the electrochemical bases of mitochondrial dysfunction by measuring ETC activity and its regulation by NAC	69 SLE patients and 37 healthy donors	There is increased O_2_ consumption through mitochondrial ETC complex I in SLE lymphocytes that is inhibited by NAC, which may have therapeutic potential by reducing oxidative stress	^[22]^
Comprehensive metabolome analyses reveal *N*-acetylcysteine responsive accumulation of kynurenine in systemic lupus erythematosus: implications for activation of the mechanistic target of rapamycin (case study)	2015	Humans	Perform a quantitative metabolome profiling of peripheral blood lymphocytes of SLE patients and compare to those treated with NAC	36 SLE patients enrolled in double-blind placebo-controlled treatment trial with NAC	Kynurenine was the top predictor of NAC effect in SLE and it stimulates mTOR activity. NAC significantly reduced kynurenine levels. There is a dominant impact on the PPP that reflect greater demand for nucleotides and oxidative stress	^[19]^

ADHD: attention-deficit/hyperactivity disorder; BILAG: British Isles Lupus Assessment Group; ETC: Electron Transport Chain; mTOR: mechanistic target of rapamycin; NAC: *N*-acetylcysteine; SLE: systemic lupus erythematosus; SLEDAI: SLE Disease Activity Index.

Direct blockade of mTOR with sirolimus also exerts promising clinical efficacy and moderated mitochondrial oxidative stress in patients ^[23]^ and mice with SLE ^[24,25]^. These findings implicated a pro-inflammatory, positive feedback loop between mTOR activation and mitochondrial dysfunction in SLE.

Another study by Doherty et al ^[22]^ was done also on the finding that SLE patients’ PBL show mitochondrial dysfunction and oxidative stress. The aim of this study was to determine the electrochemical basis of mitochondrial dysfunction by measuring the electron transport chain (ETC) activity and its regulation by NAC. Seven SLE patients, 11 healthy donors, and 10 non-lupus inflammatory arthritis donors constituted the study population. The result of this study showed that lupus PBLs have increased oxygen consumption through mitochondrial ETC complex I, which is the main source of oxidative stress in SLE, and this complex is inhibited by NAC ^[22]^.

One study done by Garcia et al ^[21]^ used the Attention-Deficit and Hyperactivity Disorder (ADHD) Self-Reported Scale (ASRS) to assess 49 patients with SLE and 46 matched healthy control subjects. Twenty-four of the patients with lupus were randomized to be given either NAC at a dose of 2.4 g/day, or NAC at a dose of 4.8 g/day or just placebo. It was found that ASRS scores were increased in patients with SLE compared with control subjects, and that this score was reduced in SLE patients treated with NAC ^[21]^.

The effect of NAC and atorvastatin on the endothelial dysfunction in patients with SLE was assessed using 32 SLE patients along with 10 healthy control subjects who were age and sex-matched. The dose of NAC used was 600 mg 3 times a day for 2 weeks. Results showed a reduction in the stiffness index and in the reflection index in the NAC-treated group suggesting improvement and endothelial dysfunction, which is associated with decreased incidence of cardiovascular and cerebrovascular accidents ^[20]^. Figure 1 below summarizes the above findings.

**Figure 1.**
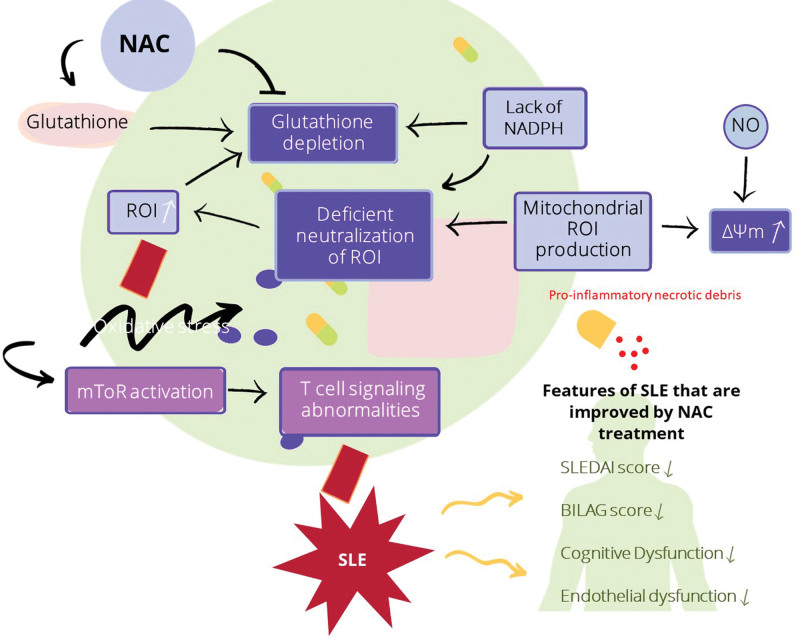
NAC beneficial effects on molecular and organismal levels. Exposure to NO and/or depletion of glutathione causes MHP and oxidative stress. The glutathione precursor NAC replenishes glutathione and prevents MHP. mTOR is a sensor of MHP and causes alteration of intracellular signal transduction and T-cell lineage specification leading to autoantibody formation and subsequent development of SLE. mTOR is also activated outside the immune system and it thus mediates end-organ damage, including the kidney and the liver. NAC treatment has had therapeutic benefits in SLE as measured by diminished SLEDAI and BILAG scores and fatigue and improved cognitive dysfunction on the ADHD Self-Reported Scale. ADHD, attention-deficit/hyperactivity disorder; BILAG, British Isles Lupus Assessment Group; MHP, mitochondrial hyperpolarization; mTOR, mechanistic target of rapamycin; NAC, *N*-acetylcysteine; SLE, systemic lupus erythematosus; SLEDAI, SLE Disease Activity Index.

Regarding reported side effects with NAC, there is inadequate measuring and reporting of the side effects of NAC in trials. Although unclear, there has been a trend of increasing side effects with increasing dosages of NAC ^[26]^. Some of the side effects encountered with the use of oral NAC are nausea, bloating, and bad taste ^[9]^. In addition to this, another limitation of the use of NAC is the oral drug bioavailability: oral administration is reported to lead to NAC concentrations below 15 μM in the circulation whereas intravenous injections of NAC achieve serum concentrations between 100 and 1500 μM ^[27]^. To improve lipophilicity of NAC, an amide derivative, *N*-acetylcysteine amide (NACA) has been synthesized, which appears to have similar membrane permeability and antioxidant properties ^[28]^. Moreover, NAC exerts anticoagulant and antiplatelet properties, and therefore, its use in patients with bleeding disorders or blood thinners is relatively contraindicated. Along this line, the concurrent use of NAC in patients on nitroglycerin should be avoided, since this combination may cause hypotension ^[29]^.

NAC dosing varies significantly with various clinical studies and doses of 1200 mg daily or more are usually required to be clinically relevant ^[28]^. Orally, it undergoes rapid intestinal absorption and metabolism by the liver, which directs most of the cysteine released toward GSH synthesis. Intravenous administration allows rapid delivery of high concentrations of NAC due to the absence of the first-pass intestinal and hepatic metabolism and that is why it is the intravenous route used for the treatment of paracetamol overdose ^[30]^.

In conclusion, the mechanism of NAC in the treatment of SLE seems to be associated with its role in the oxidative stress. A recently published 36-week open-label clinical trial was conducted in 8 older adults (OAs) and 8 young adults (YAs). OAs were studied after GlyNAC (combination of glycine and NAC) supplementation for 24 weeks, and GlyNAC withdrawal for 12 weeks. GlyNAC supplementation for 24 weeks in OAs corrected RBC-GSH (glutathione) deficiency, oxidative stress, and mitochondrial dysfunction; and improved inflammation, endothelial dysfunction, insulin-resistance, genomic-damage, cognition, strength, gait-speed, and exercise capacity; and lowered body-fat and waist-circumference ^[31]^.

The above-mentioned SLE-related studies have shown promising results with NAC use especially considering its relative safety compared to other existing, biological or conventional, immunosuppressive therapies, which invariably predispose to infections, a leading cause of death in patients with SLE. Currently, a phase II trial is under way to evaluate the tolerance and effect of NAC in SLE patients, and assess SLEDAI, BILAG, Fatigue Assessment Scale, Patient Reported Outcomes Measurement Information System, ASRS, prednisone use, liver and bone marrow function as secondary outcomes. Similar to rapamycin ^[32]^, via blockade of mitochondrial oxidative stress and mTOR ^[10,33]^, NAC may have the premise to expand antiviral CD8 T cells and reverse infections ^[8]^ and thus potentially expand life-span in SLE ^[34]^. We are currently conducting a double-blind placebo-controlled trial of 12-month duration which should provide further information on safety and efficacy of NAC in SLE (ClinicalTrials.gov Identifier: NCT00775476).

## Conflicts of interest

The authors declare that they have no conflicts of interest.

## Funding

This work was supported in part by grants R01AI072648, R01AI122176, and U01AR076092 from the National Institutes of Health, the Phillips Lupus and Autoimmunity Center of Excellence, and the Central New York Community Foundation.

## References

[1] Fava A Petri M. Systemic lupus erythematosus: diagnosis and clinical management. J Autoimmun. 2019;96:1–13. doi: 10.1016/j.jaut.2018.11.001. 30448290 10.1016/j.jaut.2018.11.001 PMC6310637

[2] Perl A. Systems biology of lupus: mapping the impact of genomic and environmental factors on gene expression signatures, cellular signaling, metabolic pathways, hormonal and cytokine imbalance, and selecting targets for treatment. Autoimmunity. 2010;43:32–47. doi: 10.3109/08916930903374774. 20001421 10.3109/08916930903374774 PMC4020422

[3] Wallace DJ. The evolution of drug discovery in systemic lupus erythematosus. Nat Rev Rheumatol. 2015;11:616–20. doi: 10.1038/nrrheum.2015.86. 26122951 10.1038/nrrheum.2015.86

[4] Tsokos GC Lo MS Reis PC. New insights into the immunopathogenesis of systemic lupus erythematosus. Nat Rev Rheumatol. 2016;12:716–30. doi: 10.1038/nrrheum.2016.186. 27872476 10.1038/nrrheum.2016.186

[5] Tipton CM Fucile CF Darce J. Diversity, cellular origin and autoreactivity of antibody-secreting cell population expansions in acute systemic lupus erythematosus. Nat Immunol. 2015;16:755–65. doi: 10.1038/ni.3175. 26006014 10.1038/ni.3175 PMC4512288

[6] Gergely PJ Grossman C Niland B. Mitochondrial hyperpolarization and ATP depletion in patients with systemic lupus erythematosus. Arthritis Rheum. 2002;46:175–90. 11817589 10.1002/1529-0131(200201)46:1<175::AID-ART10015>3.0.CO;2-H PMC4020417

[7] Banki K Hutter E Gonchoroff N. Elevation of mitochondrial transmembrane potential and reactive oxygen intermediate levels are early events and occur independently from activation of caspases in Fas signaling. J Immunol. 1999;162:1466–79. 9973403 PMC4020419

[8] Chen PM Katsuyama E Satyam A. CD38 reduces mitochondrial fitness and cytotoxic T cell response against viral infection in lupus patients by suppressing mitophagy. Sci Adv. 2022;8:eabo4271. doi: 10.1126/sciadv.abo4271. 35704572 10.1126/sciadv.abo4271 PMC9200274

[9] Lai Z-W Hanczko R Bonilla E. *N*-acetylcysteine reduces disease activity by blocking mTOR in T cells of lupus patients. Arthritis Rheum. 2012;64:2937–46. doi: 10.1002/art.34502. 22549432 10.1002/art.34502 PMC3411859

[10] Perl A. Mechanistic target of rapamycin pathway activation in rheumatic diseases. Nat Rev Rheumatol. 2016;12:169–82. doi: 10.1038/nrrheum.2015.172. 26698023 10.1038/nrrheum.2015.172 PMC5314913

[11] Mao Z Tan Y Tao J. Renal mTORC1 activation is associated with disease activity and prognosis in lupus nephritis. Rheumatology. 2022;61:3830–40. doi: 10.1093/rheumatology/keac037. 35040950 10.1093/rheumatology/keac037 PMC9608003

[12] Oaks Z Winans T Caza T. Mitochondrial dysfunction in the liver and antiphospholipid antibody production precede disease onset and respond to rapamycin in lupus-prone mice. Arthritis Rheumatol. 2016;68:2728–39. doi: 10.1002/art.39791. 27332042 10.1002/art.39791 PMC5083168

[13] Stathopoulou C Nikoleri D Bertsias G. Immunometabolism: an overview and therapeutic prospects in autoimmune diseases. Immunotherapy. 2019;11:813–29. doi: 10.2217/imt-2019-0002. 31120393 10.2217/imt-2019-0002

[14] Mokhtari V Afsharian P Shahhoseini M. A review on various uses of *N*-Acetyl cysteine. Cell J. 2017;19:11–17. doi: 10.22074/cellj.2016.4872. 28367412 10.22074/cellj.2016.4872 PMC5241507

[15] Suwannaroj S Lagoo A Keisler D. Antioxidants suppress mortality in the female NZB × NZW F1 mouse model of systemic lupus erythematosus (SLE). Lupus. 2001;10:258–65. doi:10.1191/096120301680416940. 11341102 10.1191/096120301680416940

[16] Shi D Li X Chen H. High level of reactive oxygen species impaired mesenchymal stem cell migration via overpolymerization of F-actin cytoskeleton in systemic lupus erythematosus. Pathol Biol. 2014;62:382–90. doi: 10.1016/j.patbio.2014.07.009. 25239279 10.1016/j.patbio.2014.07.009

[17] Wang H Wang G Liang Y. Redox regulation of hepatic NLRP3 inflammasome activation and immune dysregulation in trichloroethene-mediated autoimmunity. Free Radic Biol Med. 2019;143:223–31. doi: 10.1016/j.freeradbiomed.2019.08.014. 31419475 10.1016/j.freeradbiomed.2019.08.014 PMC6848782

[18] Wang H Wang G Banerjee N. Aberrant gut microbiome contributes to intestinal oxidative stress, barrier dysfunction, inflammation and systemic autoimmune responses in MRL/lpr mice. Front Immunol. 2021;12:651191. doi: 10.3389/fimmu.2021.651191. 33912174 10.3389/fimmu.2021.651191 PMC8071869

[19] Perl A Hanczko R Lai Z-W. Comprehensive metabolome analyses reveal *N*-acetylcysteine-responsive accumulation of kynurenine in systemic lupus erythematosus: implications for activation of the mechanistic target of rapamycin. Metabolomics. 2015;11:1157–74. doi: 10.1007/s11306-015-0772-0. 26366134 10.1007/s11306-015-0772-0 PMC4559110

[20] Kudaravalli J. Improvement in endothelial dysfunction in patients with systemic lupus erythematosus with *N*-acetylcysteine and atorvastatin. Ind J Pharmacol. 2011;43:311–15. doi: 10.4103/0253-7613.81511. 10.4103/0253-7613.81511 PMC3113385 21713097

[21] Garcia RJ Francis L Dawood M. Attention deficit and hyperactivity disorder scores are elevated and respond to NAC treatment in patients with SLE. Arthritis Rheum. 2013;65:1313–18. doi: 10.1002/art.37893. 23400548 10.1002/art.37893 PMC4034122

[22] Doherty E Oaks Z Perl A. Increased mitochondrial electron transport chain activity at complex I is regulated by *N*-acetylcysteine in lymphocytes of patients with systemic lupus erythematosus. Antioxid Redox Signal. 2014;21:56–65. doi: 10.1089/ars.2013.5702. 24673154 10.1089/ars.2013.5702 PMC4048573

[23] Berod L Friedrich C Nandan A. De novo fatty acid synthesis controls the fate between regulatory T and T helper 17 cells. Nat Med. 2014;20:1327–33. doi: 10.1038/nm.3704. 25282359 10.1038/nm.3704

[24] Warner LM Adams LM Sehgal SN. Rapamycin prolongs survival and arrests pathophysiologic changes in murine systemic lupus erythematosus. Arthritis Rheum. 1994;37:289–97. doi: 10.1002/art.1780370219. 8129783 10.1002/art.1780370219

[25] Caza TN Fernandez D Talaber G. HRES-1/RAB4-mediated depletion of DRP1 impairs mitochondrial homeostasis and represents a target for treatment in SLE. Ann Rheum Dis. 2014;73:1887–97. doi: 10.1136/annrheumdis-2013-203794. 10.1136/annrheumdis-2013-203794 PMC4047212 23897774

[26] Rhodes K Braakhuis A. Performance and side effects of supplementation with *N*-acetylcysteine: a systematic review and meta-analysis. Sports Med. 2017;47:1619–36. doi: 10.1007/s40279-017-0677-3. 28102488 10.1007/s40279-017-0677-3

[27] Pedre B Barayeu U Ezerina D. The mechanism of action of *N*-acetylcysteine (NAC): The emerging role of H_2_S and sulfane sulfur species. Pharmacol Ther. 2021;228:107916. 34171332 10.1016/j.pharmthera.2021.107916

[28] Paul M Thushara RM Jagadish S. Novel sila-amide derivatives of *N*-acetylcysteine protects platelets from oxidative stress-induced apoptosis. J Thromb Thrombolysis. 2017;43:209–16. doi: 10.1007/s11239-016-1450-4. 27804000 10.1007/s11239-016-1450-4

[29] Schwalfenberg GK. *N*-Acetylcysteine: a review of clinical usefulness (an old drug with new tricks). J Nutr Metab. 2021;2021:9949453. doi: 10.1155/2021/9949453. 34221501 10.1155/2021/9949453 PMC8211525

[30] Dos Santos TM Graciliano NG Moura FA. *N*-Acetylcysteine (NAC): impacts on human health. Antioxidants. 2021;10:967. doi: 10.3390/antiox10060967. 34208683 10.3390/antiox10060967 PMC8234027

[31] Kumar P Liu C Hsu JW. Glycine and *N*-acetylcysteine (GlyNAC) supplementation in older adults improves glutathione deficiency, oxidative stress, mitochondrial dysfunction, inflammation, insulin resistance, endothelial dysfunction, genotoxicity, muscle strength, and cognition: results of a pilot clinical trial. Clin Transl Med. 2021;11:e372. doi: 10.1002/ctm2.372. 33783984 10.1002/ctm2.372 PMC8002905

[32] Lai Z Kelly R Winans T. Sirolimus in patients with clinically active systemic lupus erythematosus resistant to, or intolerant of, conventional medications: a single-arm, open-label, phase 1/2 trial. Lancet. 2018;391:1186–96. doi: 10.1016/S0140-6736(18)30485-9. 29551338 10.1016/S0140-6736(18)30485-9 PMC5891154

[33] Piranavan P Bhamra M Perl A. Metabolic targets for treatment of autoimmune diseases. Immunometabolism. 2020;2:e200012. doi: 10.20900/immunometab20200012. 32341806 10.20900/immunometab20200012 PMC7184931

[34] Mannick JB Morris M Hockey HU. TORC1 inhibition enhances immune function and reduces infections in the elderly. Sci Transl Med. 2018;10:eaaq1564. 29997249 10.1126/scitranslmed.aaq1564

